# Biosorption of diesel and lubricating oil on algal biomass

**DOI:** 10.1007/s13205-012-0056-6

**Published:** 2012-03-25

**Authors:** Praveen Kumar Mishra, Suparna Mukherji

**Affiliations:** Centre for Environmental Science and Engineering (CESE), Indian Institute of Technology (Bombay), Powai, Mumbai, 400076 India

**Keywords:** Sorption isotherms, Sorption capacity, Sorption rate, Hydrocarbon sorption, Cyanobacteria

## Abstract

Algae are widely used as biosorbent for the sorption of heavy metals, however sorption of oil on algae has not been explored. Algae in marine and fresh water environment may affect the fate and transport of spilled oil. Sorption of diesel and lubricating oil was studied using dead biomass of *Spirulina* sp. and *Scenedesmus abundans*. The rate and extent of sorption was studied in well mixed batch systems containing oil (0.1–2 %, v/v) and biomass (0.1 %) suspended in water. Sorption of diesel on *Spirulina* sp. was instantaneous. Rate limited sorption on algal biomass could be explained by the pseudo-second order model and equilibrium was established within 12 days. The Freundlich and Langmuir model could provide adequate fit for diesel sorption on algae but not for lubricating oil. A three parameter model, the Sips model, provided good fit for all the experimentally generated isotherms and yielded maximum sorption capacity of diesel and lubricating oil in the range of 12–14 g/g. Oil sorption on algae was comparable to other spill clean-up sorbents. Algae can be used for development of low cost sorbents and can facilitate removal of oil from contaminated water. Sorption is affected both by the type of oil and type of algae. This is the first study illustrating that the additives present in lubricating oil not only reduces the sorption rate but also alters sorption equilibrium such that two-parameter isotherms are inadequate.

**Electronic supplementary material:**

The online version of this article (doi:10.1007/s13205-012-0056-6) contains supplementary material, which is available to authorized users.

## Introduction

Oil spills in the aquatic environment is a major environmental hazard and is reported to cause death of sea birds and other aquatic animals. Although spilled oil is primarily removed by controlled burning, dredging, dispersants, oil booms, skimmers and vacuum techniques (Ghannam and Chaalal 2003), sorption on spill clean-up sorbents is often used for containing oil spills. The spill clean-up sorbents have oil sorption capacity ranging from 0.26 to 86 g/g, depending on the type of sorbent and on the type and initial concentration of oil (*C*_o_, Moazed and Viraraghavan 2005). Organic sorbents derived from plants are often preferred over inorganic and synthetic sorbents since they are more readily biodegradable and typically have higher oil sorption capacity. The diesel sorption capacity of spill sorb and peat sorb containing 54 and 90 % organic matter was 1.18 and 4.35 g/g, respectively, for *C*_o_ range of 2–50 g/L (Biswas et al. 2005). Crude oil sorption on saw dust and oleic acid grafted sawdust was in the range of 4.23–6.4 g/g for *C*_o_ range of 2–14.5 g/L (Banerjee et al. 2006). Most studies with spill clean-up sorbents have not depicted variation in oil loading (g/g) with variation in aqueous phase concentration (g/L) at equilibrium. Use of such sorption isotherms can facilitate effective comparison across sorbents by fitting the experimentally generated isotherms with isotherm models.

Numerous studies have reported good sorption of contaminants on biomass derived from algae, fungi and bacteria. Biosorption is reported for a wide range of pollutants, i.e., heavy metals, phenolic compounds, dyes, and aromatic hydrocarbons (Aksu and Tezer 2005; Beolchini et al. 2006; Lei et al., 2007). It is widely recognized that algae can serve as low cost sorbents that can be applied for removal of various toxic heavy metal ions from aqueous solution containing less than 100 mg/L of heavy metal ions. Large amounts of algal biomass can be generated economically in open ponds and ditches or in large scale engineered bioreactors. Algae does not require organic carbon source for growth, hence, they can be cultivated in a cost effective and reliable manner using light. Algae growing phototropically can utilize carbon dioxide from the atmosphere or inorganic carbon present in water (H_2_CO_3_*, HCO_3_^−^ and CO_3_^2−^). Some algae, primarily blue-green algae (cyanobacteria) can also grow mixotrophically using both organic and inorganic carbon sources. Cyanobacteria are also known to grow effectively under low nutrient conditions. Thus, a reliable supply of algal biomass can be generated at low cost and there is much scope for utilization of algae as biosorbent due to their high surface area to volume ratio and due to the nature of their cell wall.

Researchers have focused on biosorption of metals on both green algae and cyanobacteria, such as, chromium (VI) sorption on *Spirogyra* sp*.* (Gupta et al. 2001); *Nostoc muscorum* (Gupta and Rastogi 2008) and *Oedogonium hatei* (Gupta and Rastogi 2009); cadmium sorption on *Spirulina* (Doshi et al. 2007) and sorption of copper, nickel and chromium (VI) on *Scenedesmus obliquus* and *Synechocystis* sp. (Çetinkaya et al. 1999). These studies have evaluated sorption kinetics and sorption equilibrium and have evaluated goodness of fit to various kinetic models (pseudo first order and pseudo second order model) and isotherm models. Both the sorption kinetic models have been widely applied to metal sorption data. In most cases metal sorption on algae is reported to be a fast process ranging from a few minutes to several hours. The Freundlich isotherm and Langmuir isotherm have been reported to provide good fit to metal sorption data derived from batch equilibrium studies. Desorption of sorbed heavy metal ions for promoting reuse of sorbent has also been explored and found to be feasible with proper choice of regenerant. Researchers have also attempted to decipher the sorption mechanism of various metal ions on algal biomass. Sorption has been correlated with the type of functional groups present on the algal cell wall as determined through FTIR studies (Çetinkaya et al. 1999; Doshi et al. 2007; Gupta and Rastogi 2008 and 2009). The contribution of physical sorption is reported to be much lower than that of chemisorption, where the latter primarily occurs through ion exchange in a pH dependent manner. Chojnacka et al. (2005) revealed that the growth conditions of *Spirulina* sp. (light intensity and glucose concentration determining, phototrophic, mixotrophic and autotrophic growth) significantly affected the relative abundance of various surface functional groups (carboxyl, phosphate, hydroxyl and amine) which in turn had a significant impact on biosorption of various heavy metal ions. Sorption of Cr^3+^, Cd^2+^ and Cu^2+^ ions on *Spirulina* sp. increased as pH increased due to deprotonation of the surface functional groups and development of negative charge on the surface. Over the pH range 3–9, the carboxyl and phosphate groups had maximum impact on sorption through ion exchange. In contrast, sorption of Cr(VI) on various algae and cyanobacteria was reported to be maximum in the pH range 2–3 since Cr(VI) exists as negatively charged species, i.e., chromate and dichromate and at higher pH values sorption is hindered due to electrostatic repulsion (Gupta et al. 2001; Gupta and Rastogi 2008, 2009). Sorption of Cr(VI) on *Nostoc muscorum* was found to be favored at elevated temperatures. Based on change in Langmuir isotherm parameters with change in temperature, sorption was found to be endothermic and spontaneous. The solution ionic strength is also reported to impact sorption of heavy metal ions on algae (Beolchini et al. 2006).

Although algae has been widely used as biosorbent for the sorption of heavy metals very limited research has been conducted for sorption of organic pollutants on algae. It is widely recognized that most hydrophobic organic compounds (HOCs) tend to bioconcentrate on algae, fish and other aquatic organisms, however, sorption of HOCs on algae has not been quantified as extensively as for heavy metals. Some researchers have reported sorption of organic micropollutants, such as, chlorobenzenes on *Anabaena* and *Scenedesmus* and sorption of low concentration of fluoranthene and pyrene on various microalgae (Koelmans et al. 1995; Lei et al. 2007). Sorption of chlorobenzenes was found to be essentially complete within 24 h and could be represented in terms of linear isotherms, characterized by a single parameter, i.e., organic carbon normalized partition coefficient, *K*_oc_. However, various researchers have demonstrated that sorption of HOCs is also nonlinear and needs to be described by two-parameter Langmuir or Freundlich isotherms when a sufficiently large concentration range is considered (Weber and Digiano 1996). Moreover, unlike metal sorption, HOC sorption kinetics is typically slow, with a rapid phase followed by a slow phase, hence it is important to conduct batch sorption studies over sufficiently long time periods or several days in the range of 10–30 days (Huang and Weber 1997). Also, while several HOCs are inherently volatile and are characterized by large Henry’s constant yet others, such as high MW polynuclear aromatic hydrocarbons (PAHs), are highly hydrophobic and tend to sorb strongly to glassware. This necessitates the use of controls in sorption experiments with HOCs for estimation of abiotic losses while such controls are rarely incorporated for determining sorption of heavy metals (Lion et al. 1990; Weber and Digiano 1996). Oil is composed of a multitude of HOCs existing in a high concentration in a non aqueous phase liquid (NAPL). Algae from marine and fresh water environment may have a special relevance for development of low cost sorbents for removal of oil. Interaction of algae with oil is also likely to affect the fate and transport of spilled oil in the environment and the fate of oil in oily wastewater treatment studies involving algae where significant accumulation of oil may occur on biomass (Chavan and Mukherji 2008). The objective of this study was to determine the rate and extent of sorption of diesel and lubricating oil on dead algal cultures, *Spirulina* sp. and *Scenedesmus abundans*. Sorption isotherms were generated by treating oil suspended in water as analogous to dissolved contaminants. The impact of pH, background ionic strength and temperature on biosorption of oil was also determined.

## Materials and methods

### Generation and characterization of biosorbent

*Spirulina* sp., a cyanobacteria and *Scenedesmus abundans*, a fresh water green algae, were obtained from a culture collection (NCIM 5143 and NCIM 2897, respectively, NCIM, NCL, Pune, India). Algae was cultured in a 3 L Haffkin flask containing 1.5 L of NCIM recommended nutrient medium and incubated at 28 °C under illumination of 1,500 lux provided with an incandescent lamp operated at L:D cycle of 18:6. The biosorbent was harvested by centrifugation, washed thrice, autoclaved, dried, powdered and sieved to obtain particle size less than 0.5 mm. The sorbent was stored in an air tight container in a dry place at room temperature. The autoclaving and drying steps ensured that the algal culture was inactivated, such that removal of oil from the aqueous phase was solely due to biosorption and not due to metabolic activity. The biosorbents were characterized based on BET and Langmuir surface area and pore size based on liquid nitrogen dry sorption method in a BET surface area analyzer (Micromeritics, USA). The pH at point of zero charge (pH_pzc_) values were estimated using the pH drift method (Lopez-Ramon et al. 1999). The functional groups present on the cell wall of algal samples, *Spirulina* and *Scendesmusabundans*, were analyzed using FTIR spectroscopy (Vertex 80 FTIR System, Bruker, Germany). A small amount of algal biomass in dry powder form was mixed with KBr and the mixed powder was pressed to form a pellet. The spectrum (transmission mode) was obtained in the range of 4,000–500 cm^−1^ at a resolution of 0.2 cm^−1^ at room temperature.

### Batch studies for biosorption of oil

Lubricating oil (IOCL, Bhandup, Mumbai, India) and diesel (petrol station in Bhandup, Mumbai, India) was first artificially weathered in an open container for 48 h. In the kinetic studies a fixed concentration of oil [*C*_o_ = 0.5 % (v/v) for *Spirulina sp*. and 1 % (v/v) for *S. abundans*] and a fixed dose of biomass (0.1 %, w/v) were used. The pH, temperature and concentration of NaNO_3_ used as background electrolyte were 7, 30 °C and 0.01 M, respectively. The kinetic study was performed using multiple completely mixed batch reactors (CMBR, 50 mL glass serum bottle capped with Teflon lined septa) tumbled end over end at 50 rpm. For estimating residual oil in the aqueous phase, the algal biomass was removed by centrifugation (12,000 rpm for 10 min). Subsequently, oil was determined by gravimetry after extracting oil from the aqueous phase by adding dichloromethane (DCM, 1:1 v/v) and centrifuging at 10,000 rpm for 10 min (Biswal et al. 2009). Sorbed oil was calculated based on mass balance assuming same abiotic losses for the control and experimental batches. Rate limited sorption of oil on algae was fitted with the pseudo-second order kinetic model (Eq. 1, Ozer et al. 2006).1 where, *q*_t_ and *q*_e_ (g/g) is the oil sorbed at time‘*t*’ (d) and at equilibrium, respectively, and *k*_2_ [(g/g)^−1^ d^−1^] is the pseudo-second order biosorption rate constant.

Batch isotherm studies under the same conditions were set-up in multiple CMBRs (50 mL) with *C*_o_ ranging from 0.1 to 2 % (v/v). Duplicate samples were extracted after providing sufficient time for achieving equilibrium. For each *C*_o_, *q*_e_ was calculated using Eq. (2) (Paikaray 2006) which accounts for abiotic losses, such as volatilization. *Ŝ* is the slope derived from a plot of concentration in the controls at equilibrium (*C*_c_) versus *C*_o_.2

The experimentally generated isotherms (*q*_e_ vs. *C*_e_ data, expressed in g/g and g/L, respectively) were fitted using various two-parameter (Langmuir, eqn. 3 and Freundlich, Eq. 4) and three-parameter isotherm models (Redlich-Peterson, Eq. 5 and Sips model (Langmuir–Freundlich model), Eq. 6) (Kumar and Porkodi 2006, Ho et al. 2002). The isotherm parameters for each model were determined using nonlinear regression performed using SYSTAT 10.2 by Gauss–Newton method where the best fit parameter estimates were obtained by minimizing the sum of squared residuals.3456

where, the parameters, *Q*_a_^o^ (g/g), K_F_ (g/g), *A*_RP_ (L/g)_,_*Q*_*m*_^*s*^ (g/g) are the terms related to maximum sorption capacity and *b* (L/g), *n*, b_RP_ (L/g), *k*_*s*_ (L/g) are the terms related to sorption energetics in the various models. When ‘*g*’ and ‘*m*_*s*_’ attains a value of unity in Eqs. 5 and 6, respectively, these expression reduces to the Langmuir isotherm.

The effect of ionic strength (0.01–1 M NaNO_3_), pH (2–12) and temperature (20–45 °C), on sorption was tested using a fixed initial concentration of oil of 1 % (v/v) with other conditions remaining same. Sorbed oil at equilibrium was estimated using Eq. 2 using the same *Ŝ* values as determined for the equilibrium studies. Abiotic controls were also maintained to determine the variation in abiotic losses with variation in the experimental conditions. All experiments were conducted in duplicate. The error bars represent standard error and were determined based on propagation of errors for derived variables.

## Results and discussion

The yield of dry biomass was 680 and 360 mg/L, respectively for *Spirulina* sp*.* and *S. abundans* at the end of log growth phase (11 and 21 days, respectively)*.* In a batch culture, the log growth phase signifies the period when there is an exponential increase in cell count and biomass. Subsequently, conditions in the batch culture are no longer conducive for supporting growth at such a rapid rate. End of log growth phase signifies either depletion of nutrients, increase in pH to levels that inhibit growth, accumulation of toxins or shading effect (dense growth causing hindrance in penetration of light). The biosorbent prepared using *Spirulina* sp. yielded higher BET and Langmuir surface area (20.95 and 73.94 m^2^/g, respectively) compared to the biosorbent prepared with *S. abundans* (1.38 and 2.08 m^2^/g, respectively). These surface area values based on physical sorption of nitrogen gas molecules on the surface of a sorbent by fitting the BET isotherm and Langmuir isotherm to nitrogen sorption data, respectively, are widely used to characterize the surface area of sorbent. The average pore width for *Spirulina* sp. biosorbent was 73.27 (Å) while that for *S. abundans* biosorbent was 366.77 (Å). The pH_PZC_ was found to be 8.5 for *Spirulina* sp. and 7.5 for *S. abundans*. The FTIR spectra for both *Spirulina* and *S. abundans* were very similar to that reported by Doshi et al. (2007) for a *Spirulina* sp*.* (supplementary material). The identity of functional groups listed in Table 1 was interpreted based on the spectra obtained and analysis of FTIR spectra for alga reported by various researchers (Gupta and Rastogi 2009; Doshi et al. 2007; Stehfest et al. 2005; Çetinkaya et al. 1999).

**Table 1:** Functional Groups based on FTIR spectra of *Spirulina* and *Scendesmus abundans*

Wave number (cm^−1^)		Functional Groups
*Spirulina* sp*.*	*Scendesmus abundans*	
3324	3403	Carboxylic/phenolic –OH stretching and stretching of NH_2_
2958, 2925, 2855	2951, 2926, 2855	–CH stretching
1652	1652	C=O stretching of amide-I from proteins
1540	1541	N–H stretching of amides from proteins; amide-II band
1456	1456	Asymmetric deformation of CH_3_ group of proteins
1404	1456	Symmetric deformation of CH_2_ and CH_3_ group of protein, stretching of C–O bond in COO^−^
1243	1252	Stretching of P=O in phospholipids and nucleic acids, stretching of =C–C=
1154, 1047	1067	C–O–C stretching of polysaccharides
	873	Plane deformation
667		
576	586	Stretching and bending modes of phosphate

**Fig. 1:**
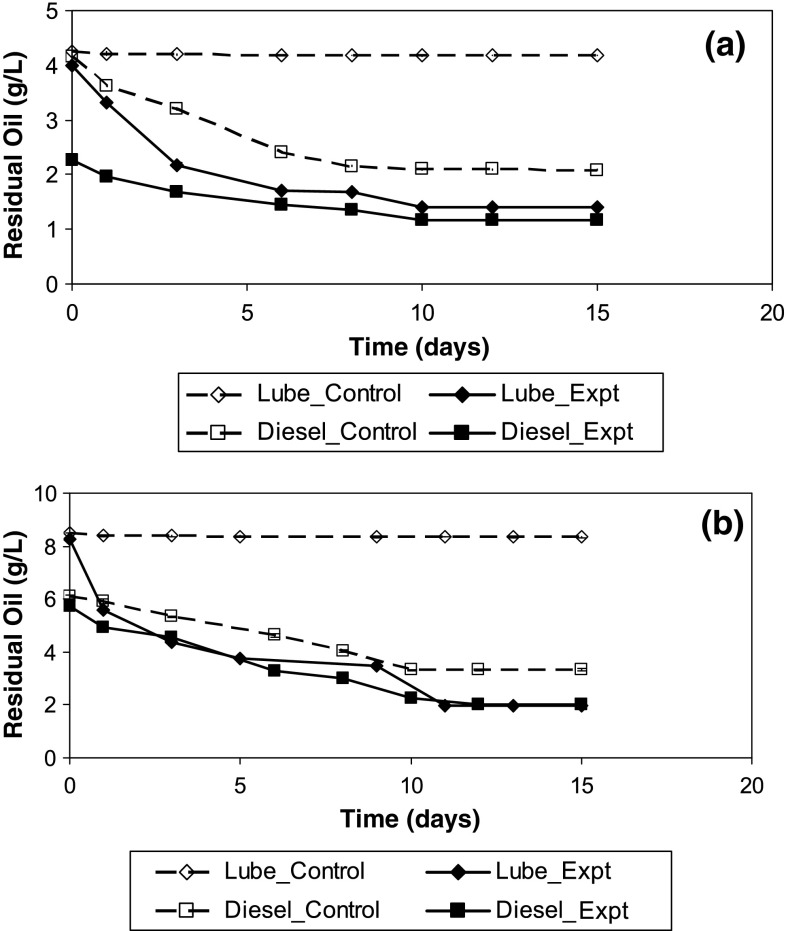
Sorption of lubricating oil and diesel on (**a**) *Spirulina* sp. (*C*_o_ = 0.5 %) and (**b**) *Scendesmus abundans* (*C*_o_ = 1 %) over a period of 16 days

**Fig. 2:**
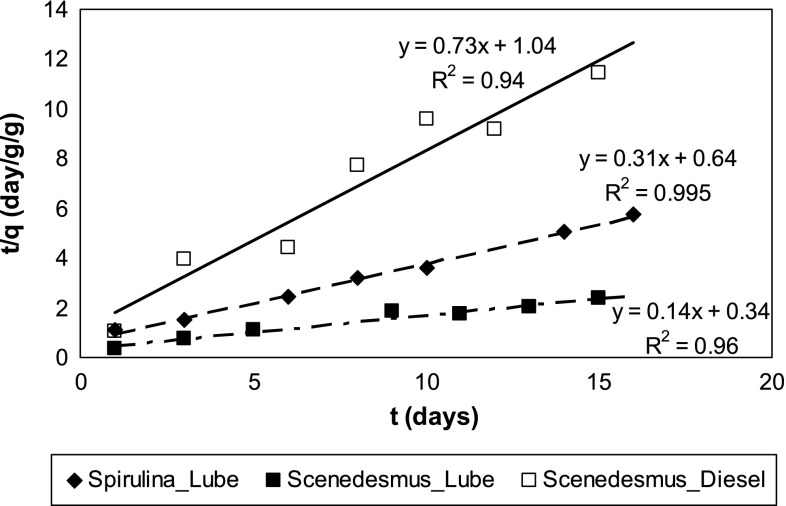
Rate limited sorption of diesel and lubricating oil on algae fitted to pseudo-second order model

The controls indicated much lower extraction loss and additional abiotic loss over 16 days for lubricating oil (<4 %) than for diesel (50–60 %) (Fig. 1). The aqueous concentration profile stabilized after nearly 10–12 days. Diesel sorption on *Spirulina* sp*.* was instantaneous (Fig. 1). Rate limited sorption of oil on algae followed the pseudo-second order kinetics (Fig. 2). The faster sorption rates for diesel compared to lubricating oil on *S. abundans* is indicated by the pseudo-second order rate constant, *k*_2_, i.e., 0.51 ± 0.37 and 0.06 ± 0.02 [(g/g)^−1^ d^−1^] for diesel and lubricating oil, respectively. Lubrication oil sorption on *Spirulina* sp*.* [*k*_2_ = 0.15 ± 0.02 (g/g)^−1^d^−1^] was faster than on *S. abundans*. The kinetic studies for *Spirulina* sp. was conducted using a lower *C*_o_ (0.5 %) than for *S. abundans* (1 %), thus, at the same *C*_o_ differences in *k*_2_ values for *Spirulina* sp*.* and *S. abundans* is expected to be even greater (Aravindhan et al. 2007; Ozer et al. 2006).

The equilibrium studies were set-up for 10 days for all oil-algae combination other than for lubricating oil sorption on *S. abundans*, where 14 days equilibration time was provided. The *q*_e_ values reported were obtained using Eq. 2 which corrects for abiotic losses. Some loss of oil is expected due to sorption on glassware and due to volatilization. These losses would occur both in the controls and in the experimental flasks. If uncorrected, they would overestimate sorption on algae. This can cause large errors for volatile oils, such as, diesel. Hence, these losses were estimated based on the controls devoid of algal biosorbent set up with varying initial concentration of oil. If no loss occurs, concentration in the controls after equilibrium (*C*_c_) would be same as the initial concentration (*C*_o_) and a plot of *C*_c_ versus *C*_o_ would be a straight line with slope of unity. However, due to these losses *C*_c_ is lower than *C*_o_, and it has been reported that *C*_c_ and *C*_o_ are linearly related, with a slope of *Ŝ* (where, *Ŝ* ≤ 1), such that *C*_o_ = *C*_c_/*Ŝ*. The residual concentration observed in the experimental flask with sorbent (*C*_e_) would be equal to *C*_e_/*Ŝ* after correction for these losses, and this corrected value needs to be used for estimating sorption on algae. The lower the value of *Ŝ*, the higher is the abiotic losses due to volatilization and sorption on glassware. An alternative approach for estimating abiotic loss, assumes equal abiotic loss in both the controls and experimental batches for a fixed value of *C*_o_, however, this approach would underestimate sorption on the biosorbent.

Good linearity was observed in plots of concentration in controls (*C*_c_) at equilibrium versus initial concentration (*C*_o_) with *R*^2^ = 0.99 for both lubricating oil and diesel. The slope value (*Ŝ*) was 0.96 for lubricating oil and 0.595 for diesel. The significantly lower *Ŝ* value for diesel compared to lubricating oil maybe be expected since diesel is composed of *n*-alkanes with lower chain length (nC9–nC24) compared to lubricating oil (nC14–nC40) and is thus subjected to greater volatilization losses (Wang and Stout 2007) as was also evident from the kinetic studies. Alternatively, if *q*_e_ was estimated assuming equal abiotic losses for control and experimental batches with sorbent corresponding to any *C*_o_ value, large errors would have affected the diesel sorption estimates. Comparing the observed *q*_e_ values with those reported in the literature for similar range of initial oil concentration, algae was found to have good oil sorption potential comparable to other sorbents, such as, oleic acid grafted sawdust, spill sorb and peat sorb (Banerjee et al. 2006; Biswas et al. 2005). *Spirulina* sp*.* was found to be a better sorbent for diesel as compared to the algal consortia predominated by the cyanobacteria, *Phormidium* (Chavan 2008).

**Fig. 3:**
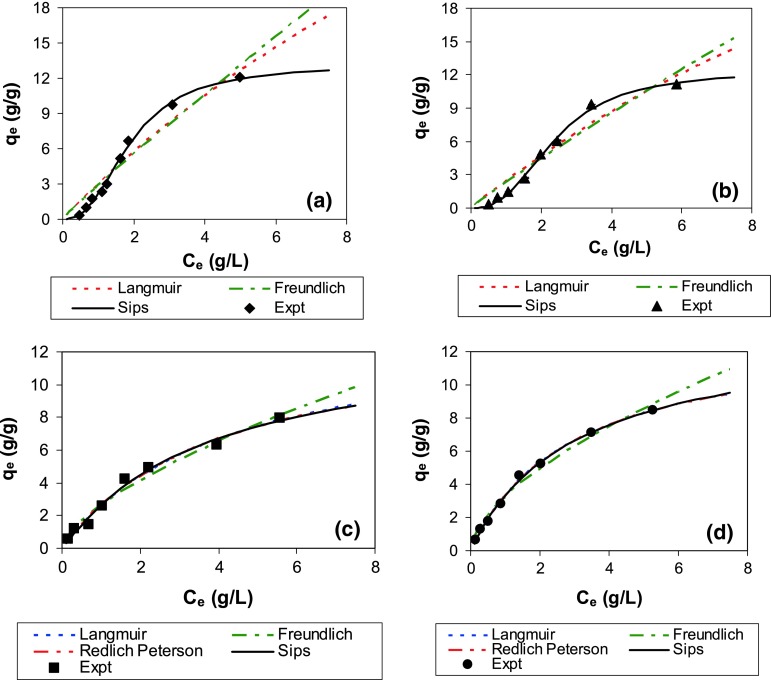
Comparison of various model fits to isotherm data for sorption of lubricating oil on (**a**) *Spirulina* sp. and (**b**) *Scenedesmus abundans* and for sorption of diesel on (**c**) *Spirulina* sp. and (**d**) *Scenedesmus abundans*

All the isotherms (Fig. 3) depicted significant nonlinearity and were thus fitted using the various two and three parameter isotherm models (2a–d) and the parameters were determined by non-linear regression (Table 2). Convergence could not be obtained for Redlich–Peterson model for lubricating oil sorption on both the algal cultures. The various two and three parameter models used yielded *R*^2^ values ≥0.9 for all other cases. Although *R*^2^ is widely used to denote significance of regressions, it does not effectively represent significance of regressions, particularly for non-linear regression. Even for linear regression, its magnitude is affected by various parameters, such as, the range of independent variable values and the number of data points (Berthouex and Brown, 1994). The high *F* value for all the regressions, however, clearly indicates that all the models are associated with high significance level (negligible *p* values → 0). However, for lubricating oil sorption on *Spirulina* sp*.* and *S. abundans*, the parameter estimates for the Langmuir model were associated with large errors and the model fit to data was also inadequate. Although the Freundlich model parameters for lubricating oil sorption on these algal cultures were not associated with such large errors, the model fits to data were found to be unsatisfactory. The Sips model provided the best fit for lubricating oil sorption on these algal cultures. The exponent ‘*m*_*s*_’ is reported to account for heterogeneity of the sorbents and interaction between sorbed layers. The ‘*m*_*s*_’ value in Sips isotherm for lubricating oil sorption was significantly higher than 1, indicating the inherent nonlinearity in this isotherm. The Sips isotherm ‘*m*_*s*_’ value is typically reported to be <1, hence, the observed values for lubricating oil sorption on both the algal cultures were contrary to expectation. Based on Sips model, the maximum sorption capacities for sorption of lubricating oil on *Spirulina* sp*.* and *S. abundans* were 13.1 and 12.4 g/g, respectively. The Langmuir and Freundlich isotherms provided good fits for diesel sorption. The exponent values in the Redlich–Peterson and Sips model were both close to unity for diesel sorption, indicating that these models essentially reduce to the two-parameter Langmuir model. Based on both Langmuir and Sips model, diesel sorption capacity on both the algal cultures were in the range of 12.5–14 g/g. Thus, the sorption capacity of both diesel and lubricating oil on these algal cultures are comparable. This is also indicated by the Freundlich model term related to sorption capacity (K_F_) which lies in the range 2.3–3.2 (g/g) for all the cases. The Freundlich exponent ‘n’ values were significantly less than unity for diesel sorption isotherms, while they were closer to unity for lubricating oil sorption isotherm. Lower ‘*n*’ values are indicative of more favorable isotherms indicating that *q*_e_ remains comparatively higher as *C*_e_ reduces (Weber and Digiano 1996). Thus, although sorption capacity for lubricating oil and diesel is comparable for these algal cultures, relatively higher loading on these biosorbents may be expected for diesel at lower *C*_e_ values. Although the BET and Langmuir surface area indicated much higher sorption potential for *Spirulina* sp. compared to *S. abundans* this was not observed for oil sorption. The BET and Langmuir surface area values were based on sorption of nitrogen gas and thus cannot adequately represent the surface area on the sorbent that is accessible for components in oil. Oil sorption is expected to be partly due to absorption/partitioning on organic matter and partly due to adsorption on the surface. Sorption of oil may thus be affected more by the organic carbon content (which is expected to be comparable for both the algal cultures) than by surface area of the sorbent. Moreover, the nature and type of functional groups on the sorbent surface may play an important role in oil sorption (Lei et al. 2007). The functional groups on the surface of both the algal cultures were very similar as revealed through the FTIR studies. Thus, comparable oil sorption capacities on both the cultures may be expected.

**Table 2:** Isotherm model parameters based on non-linear regression for sorption of lubricating oil and diesel on *Spirulina* sp*.* and *Scenedesmus abundans*

Isotherm models	Parameters	*Spirulina* sp.					*Scenedesmus abundans*				
		Mean	SE	% Error	*F*	*R* ^2^	Mean	SE	% Error	*F*	*R* ^2^
Lubricating oil											
Langmuir	*Q*_a_^o^ (g/g)	67	63	95	136	0.938	58	59	102	110	0.933
	*b* (L/g)	0.05	0.05	111			0.04	0.05	119		
Freundlich	K_F_ (g/g)	2.9	0.4	15	120	0.93	2.3	0.5	20	96	0.924
	*n*	0.94	0.12	12			0.94	0.14	15		
Sips	*Q*_*m*_^*S*^ (g/g)	13.1	0.6	5	1,045	0.995	12.4	0.7	5	870	0.995
	*k*_*S*_ (L/g)	0.52	0.03	6			0.42	0.02	6		
	*m*_*S*_	2.5	0.2	9			2.6	0.3	10		
Diesel											
Langmuir	*Q*_a_^o^ (g/g)	13.8	1.5	11	716	0.987	13.3	0.6	4	3,249	0.997
	*b* (L/g)	0.24	0.05	20			0.34	0.03	9		
Freundlich	K_F_ (g/g)	2.6	0.2	7	425	0.978	3.2	0.2	5	615	0.984
	*n*	0.65	0.05	8			0.60	0.04	7		
Redlich-Peterson	*A*_RP_ (L/g)	3.2	0.8	27	416	0.987	4.4	0.6	13	1,805	0.997
	*b*_RP_ (L/g)	0.2	0.3	128			0.3	0.2	47		
	*g*	1.1	0.5	47			1.0	0.2	17		
Sips	*Q*_m_^S^ (g/g)	12.7	3.8	30	420	0.987	13.4	1.8	13	1,805	0.997
	*k*_S_ (L/g)	0.28	0.17	60			0.33	0.09	29		
	*m*_*S*_	1.1	0.2	20			0.99	0.09	9		

*C*_e_ is expressed in g/L,*q*_e_ is expressed in g/L, and the exponent values in Freundlich, Redlich Peterson and Sips model are dimensionless

Convergence could not be obtained for Redlich Peterson model fit to the data for lubricating oil sorption on both the algal cultures

**Fig. 4:**
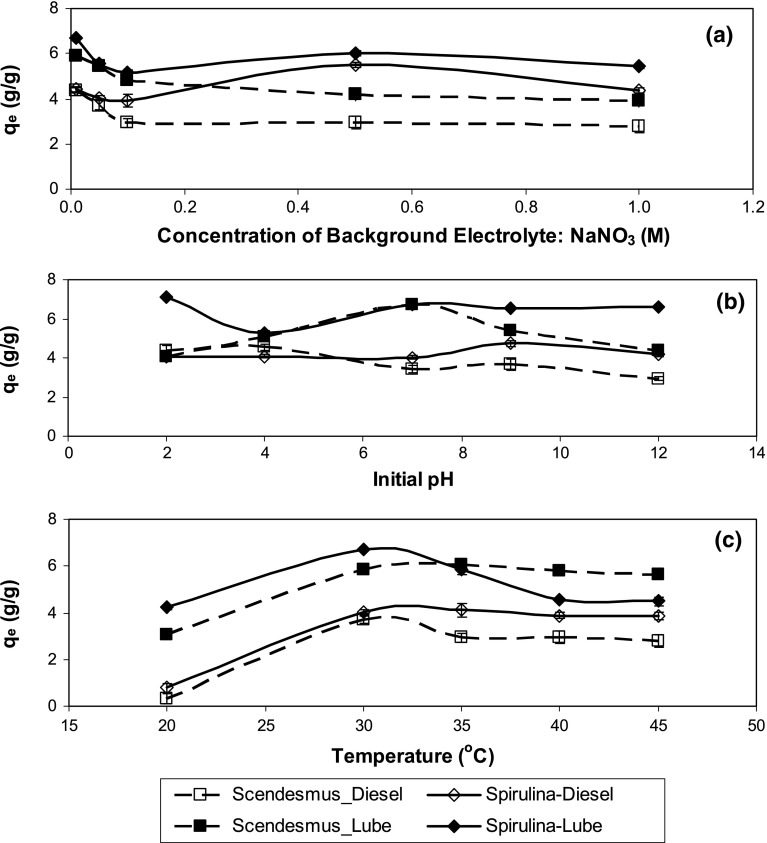
Effect of various parameters for sorption on algae (**a**) ionic strength, (**b**) pH, (**c**) temperature

Effect of various environmental parameters such as ionic strength, pH and temperature is illustrated in Fig. 4a–c. Since *Ŝ* was assumed to be unaffected by ionic strength, pH and temperature the goodness of this assumption was verified by observing variation in *C*_c_ with ionic strength, pH and temperature (supplementary material). While pH and ionic strength had no effect on *C*_c_, increasing temperature had a distinct declining trend which may cause some error in estimation of *q*_e_. In general, maximum sorption was attained at low ionic strength, at neutral pH and at 30–35 °C. The expected trend for dissolved organic solute, i.e., increase in sorption with increase in ionic strength (Chung et al. 2007) was not observed for oil sorption on algae. The trend in q_e_ variation with ionic strength was significantly affected by the type of algae. Sorption of both lubricating oil and diesel on *Spirulina* sp. was highest at the lowest ionic strength (background electrolyte, NaNO_3_, concentration of 0.01 M), decreased up to NaNO_3_ concentration of 0.1 M, increased significantly at 0.5 M and subsequently decreased again as NaNO_3_ concentration was increased to 1 M. Sorption of lubricating oil and diesel on *S. abundans* continuously decreased up to 0.5 M and 0.1 M NaNO_3_, respectively, and subsequently remained invariant as NaNO_3_ concentration was increased up to 1 M.

The pH dependence was lower for sorption of diesel than for sorption of lubricating oil on both algae. Sorption of oil on algae may be partly due to absorption/partitioning of oil on to organic matter and partly due to adsorption on to the surface of algae due to specific interactions. Absorption/partitioning of HOCs is expected to be a faster phenomenon compared to adsorption and while absorption can be explained by linear isotherms, adsorption is manifested in terms of nonlinear sorption isotherms (Weber and Digiano 1996). It is difficult to determine the relative contribution of the two processes for complex sorbates, such as, diesel and lubricating oil. While the impact of pH on absorption/partitioning may be negligible, pH may have a strong influence on adsorption. For example, sorption of heavy metals occurs primarily by adsorption and is strongly influenced by the system pH. At any pH, the surface functional groups attain positive or negative charge depending on their pK_a_ values. It has been shown that for heavy metal ions that carry a positive charge, sorption increases as pH increases over the range 2–6 due to increasing deprotonation of surface functional groups (carboxyl and phosphate groups) and development of negative charge on the surface that promotes ion exchange (Chojnacka et al. 2005). In contrast, for Cr(VI) that exist as a negatively charged species in aqueous solution, sorption decreases as pH increases over the range 2–6 due to development of negative charge on the surface that hinders chemisorption through ion exchange with surface functional groups. While sorption of oil is unlikely to occur through ion exchange, the spontaneous protonation and deprotonation of surface functional groups may still influence sorption. Hydrocarbon and oil droplets often carry negative charge in aqueous solution (Djerdjev and Beattie 2008). Thus, negative charge on the biosorbent surface at pH exceeding pH_PZC_ may hinder sorption through electrostatic repulsion and this may have an adverse impact on both absorption and adsorption processes. Moreover, pH can also impacts the surface charge on sorbates (Davis et al. 2003, Ozer et al. 2006). The surface charge on algae would depend on the protonation-deprotonation status of the various functional groups, i.e., carboxyl, hydroxyl, amides and amines, present on algae as depicted in Table 1. At a pH equal to pH_PZC_ the surface would be uncharged and sorption is expected to be maximum at this pH. In general, *q*_e_ was high at neutral pH close to pH_PZC_, however, the high *q*_e_ value for lubricating oil on *Spirulina* sp. (Fig. 4b) at pH 2 cannot be explained on this basis. Diesel sorption on both the sorbents depicted less variation with pH changes compared to lubricating oil sorption. It is possible that the contribution of absorption/partitioning is more for sorption of diesel on algae compared to sorption of lubricating oil on algae as also evident from the significantly greater nonlinearity associated with lubricating oil isotherms. The additives added to lubricating oil possibly affect its sorption behavior.

At 20 °C, diesel sorption on both algae was remarkably reduced. In all cases sorption was highest at 30–35 °C. Subsequently, sorption remained relatively constant with increase in temperature, except for lubricating oil sorption on *Spirulina* sp*.* where a sharp drop in sorption was observed as the temperature increased from 30 to 40 °C. Decrease in sorption with increase in temperature is indicative of an exothermic sorption process. Increase in temperature from 20 to 30 °C may have increased the diffusivity of oil and thus overcome mass transfer rate limitation (Ozer et al. 2006). Thus, low sorption at 20 °C may be attributed to rate limited mass transfer.

The differences in sorption observed for *Spirulina* sp. and *S. abundans* is possibly due to differences in their cell wall structure, surface properties and differences in the arrangement of cells of the prokaryotic and eukaryotic organism, respectively (Davis et al. 2003; Lei et al. 2007). The cyanobacteria, *Spirulina*, is a prokaryotic organism consisting of spiral shaped chains of cells (trichomes) enclosed in a thin sheath. It is reported to have a layered cell wall structure where a peptidoglycan layer is flanked by fibrillar layers on both sides. The eukaryote, *Scenedesmus abundans*, is a green algae, also having a layered cell wall with 2–3 distinct layers containing cellulose and pectin. It grows in colonies comprising of four cells attached side by side and has tiny projections. The difference in sorption kinetics and sorption equilibrium observed between the two types of oil is related to the differences in the nature of their constituents. Diesel is primarily composed of aliphatic hydrocarbons from C9 to C24. Lubricating oil consists of about 90 % mineral oil and 10 % additives that contribute to reduced friction, increased viscosity and corrosion resistance. Lubricating oil is also reported to contain naphthenic acids in addition to hydrocarbons. The GC chromatograms of lubricating oil is characterized by a large unresolved complex mixture (UCM) hump while that of diesel shows an abundance of distinct peaks with a very small UCM hump (Mohanty and Mukherji 2008; Biswal et al. 2009).

## Conclusions

Although algal cultures have been extensively used as sorbent for heavy metals, this is the first study reporting biosorption of oil on algae. Significant sorption of oil on algae, comparable to that of other oil spill clean-up sorbents is illustrated. Sorption rate of lubricating oil is found to be significantly lower than for diesel due to the additives present in lubricating oil. Two parameter isotherms, such as, Langmuir and Freundlich isotherms, that have been used for some oil sorption studies were adequate for diesel but not for lubricating oil. Algae can be used for development of low cost sorbents for removal of oil and can affect the fate and transport of spilled oil.

## Electronic supplementary material

Below is the link to the electronic supplementary material.

Supplementary material 1 (PDF 55 kb)

Supplementary material 2 (PDF 55 kb)
