# Supplementary figures and images for: Biosorption of diesel and lubricating oil on algal biomass
Source: 3 Biotech. 2012 Mar 25;2(4):301–10. doi: 10.1007/s13205-012-0056-6 (PMC3482444; doi:10.1007/s13205-012-0056-6)

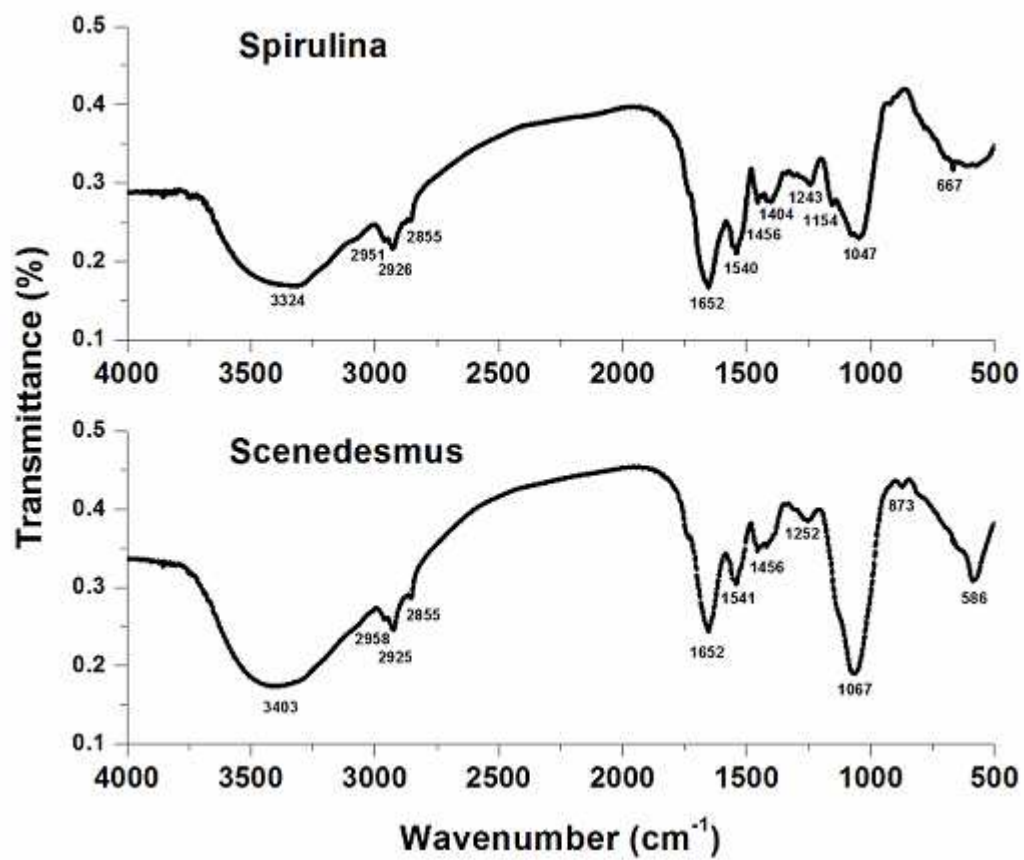

Figure S1. FTIR Spectra of *Spirulina* sp. and *Scenedesmus abundans*

Supplement: Supplementary file 1 — Supplementary material 1 (PDF 55 kb) [file 13205_2012_56_MOESM1_ESM.pdf]
