# Supplementary material for: Biosorption of diesel and lubricating oil on algal biomass
Source: 3 Biotech. 2012 Mar 25;2(4):301–10. doi: 10.1007/s13205-012-0056-6 (PMC3482444; doi:10.1007/s13205-012-0056-6)
Supplement: Supplementary file 2 — Supplementary material 2 (PDF 55 kb) [file 13205_2012_56_MOESM2_ESM.pdf]

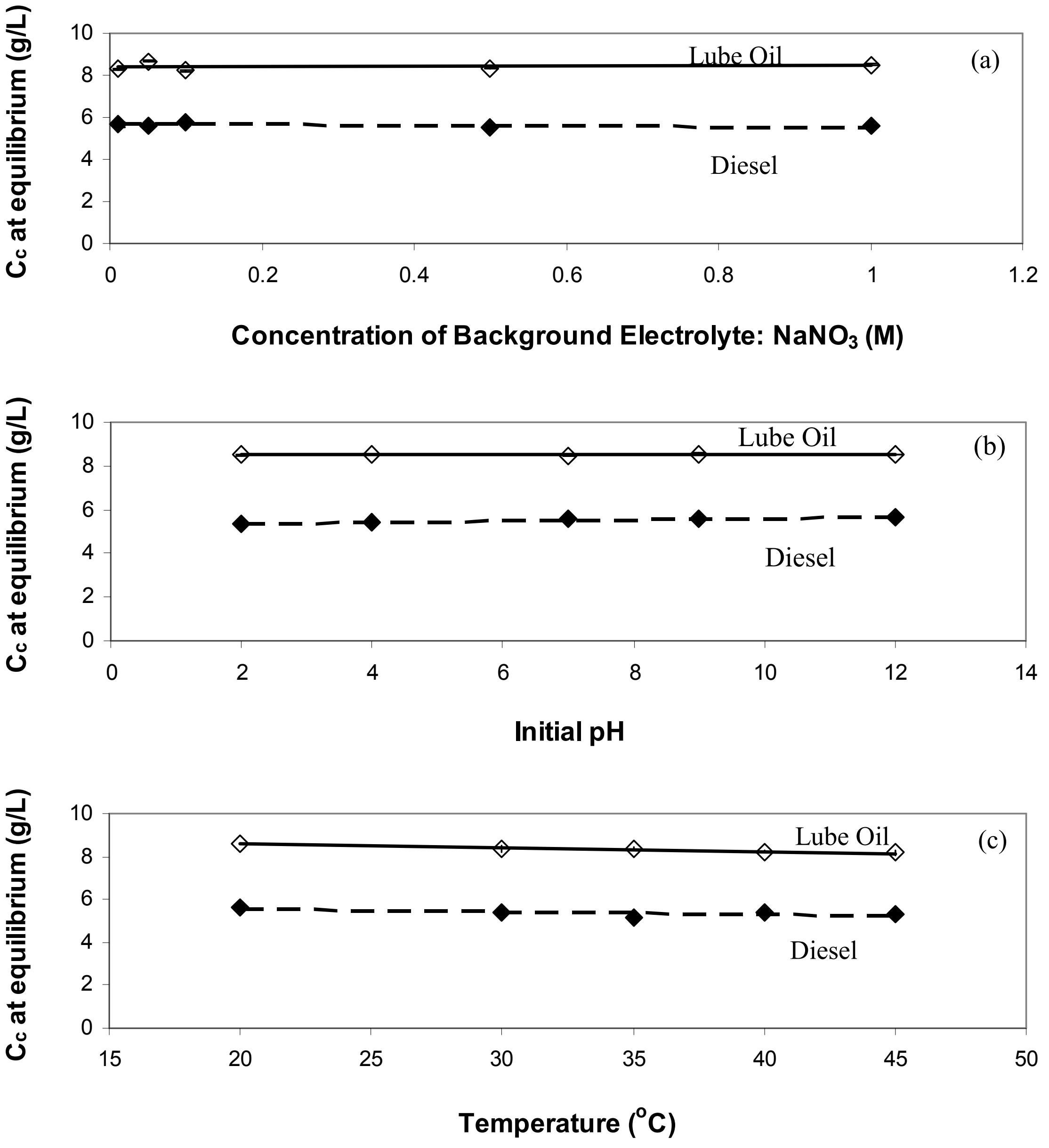

Figure S2. Concentration in the controls at equilibrium (Cc) versus (a) ionic strength, (b) pH and (c) temperature
